# Breath-holding during the Calibration Scan Improves the Reproducibility of Parallel Transmission at 7T for Human Brain

**DOI:** 10.2463/mrms.mp.2015-0137

**Published:** 2016-03-21

**Authors:** Taisuke Harada, Kohsuke Kudo, Ikuko Uwano, Fumio Yamashita, Hiroyuki Kameda, Tsuyoshi Matsuda, Makoto Sasaki, Hiroki Shirato

**Affiliations:** 1 Department of Radiation Medicine, Hokkaido University Graduate School of Medicine; 2 Department of Diagnostic and Interventional Radiology, Hokkaido University Hospital, N14 W5, Kita-ku, Sapporo, Hokkaido 060-8648, Japan; 3 Division of Ultrahigh Field MRI, Iwate Medical University; 4 MR Applications and Workflow, GE Healthcare

**Keywords:** 7T magnetic resonance imaging, parallel transmission, breath-holding, Bloch-Siegert method

## Abstract

**Purpose:**

The B0 and B1+ maps required for calculation of the radiofrequency (RF) pulse of parallel transmission (pTx) are obtained in calibration scans; however, they may be affected by respiratory motion. We aimed to compare the reproducibility of B0 and B1+ maps and gradient echo (GRE) images of the brain scanned with pTx at 7T between free-breathing (FB) and breath-holding (BH) conditions during the calibration scan.

**Methods:**

Nine healthy volunteers were scanned by 7T MRI using a two-channel quadrature head coil. In the pTx calibration scans performed with FB and BH, the B0 map was obtained from two different TE images and the B1+ map was calculated by the Bloch-Siegert method. A GRE image (gradient-recalled-acquisition in steady state) was also obtained with RF shimming and RF design of pTx with spoke method, as well as quadrature transmission (qTx). All the scans were repeated over five sessions. The reproducibility of the B0 and B1+ maps and GRE image was evaluated with region-of-interest measurements using inter-session standard deviation (SD) and coefficient of variation (CV) values. Intensity homogeneity of GRE images was also assessed with in-plane CV.

**Results:**

Inter-session SDs of B0 and B1+ maps were significantly smaller in BH (*P* < 0.01). Inter-session CVs of GRE images were significantly smaller in qTx than BH and FB (*P* < 0.01, both); however, the CVs of BH were significantly smaller (*P* < 0.01). In-plane CVs of FB and BH with RF shimming were not significantly different with qTx; however, CVs of FB and BH with RF design were significantly smaller than those of qTx (*P* < 0.05 and *P* < 0.01, respectively).

**Conclusion:**

BH could improve the reproducibility of B0 and B1+ maps in pTx calibration scans and GRE images. These results might facilitate the development of pTx in human brain at 7T.

## Introduction

The current limitation of field strength in magnetic resonance imaging (MRI) for clinical practice in Japan is 3T for clinical purposes. However, ultrahigh field (UHF) MRI [such as 7 Tesla (7T) MRI] has been used and gradually spread for research purposes. While there are many advantages of UHF MRI, such as a higher signal-to-noise ratio (SNR), higher contrast-to-noise ratio (CNR), higher spatial resolution, larger contrast of susceptibity, and higher frequency resolution in MR spectroscopy, have also disadvantages such as inhomogeneity in the static magnetic field (B0) and radiofrequency (RF) magnetic field (B1+), strong susceptibility effects, and chemical shift artifacts.^1,2^ Among these, B1+ inhomogeneity is one of the most important problems as it causes intensity inhomogeneity, low CNR. These result from B1+ focusing and standing waves because of the higher resonance frequency of protons (e.g., 300 MHz at 7T).^1,3,4^

Parallel transmission (pTx) has been developed to control the B1+ distributions, such as uniform or local excitations.^1,3,5^ The pTx system consists of multiple RF exciters and transmit coils, in which each transmit coil is simultaneously and independently driven by the RF exciter. RF shimming of the pTx system could optimize the amplitude and phase of each array element with the same waveform of the RF pulse to modify a B1+ field. In addition, specific designs of the RF waveform (RF design) have been applied to achieve better B1+ homogeneity.^6,7^

B1+ maps of each channel are required to calculate the optimal amplitude and phase of the RF pulse in RF shimming or in determining the optimal waveforms in RF design. B0 maps are also needed for RF design.^6,7^ Many methods for B1+ mapping have been reported, such as Bloch-Siegert, phase sensitive method, double angle, and actual flip angle methods.^8–11^ Among them, the Bloch-Siegert method has been reported to be an accurate and efficient phase-based method that is largely insensitive to T_1_ and T_2_. Therefore, a number of studies have used this method for B1+ mapping in UHF MRI.^11^

It has been reported that respiration can cause shifts in local resonance frequency in functional MRI, especially in the UHF,^12–15^ i.e., the phase shift occurs between inspiration and expiration due to the motion of the chest wall and local oxygen concentration changes. Therefore, respiration-induced phase fluctuations can affect B0 mapping in the calibration scan of pTx, especially for UHF such as those used in 7T MRI. This phenomenon can also affect B1+ mapping with the Bloch-Siegert method, as this technique uses phase images with two different off-resonance pulses. To our knowledge, however, there have been no studies that have examined the effect of respiratory fluctuations in pTx calibration for B0 and B1+ mapping. We hypothesized that the respiration-induced phase fluctuation could affect B0 and B1+ mapping, resulting in deterioration of the reproducibility of pTx images, which can be improved by simple breath-holding (BH). This phenomenon is potentially important for the development of a pTx system for human subjects, as it never arises in phantom studies.

The purpose of this study was to evaluate the reproducibility of B0 and B1+ maps, and gradient echo (GRE) images scanned with pTx, by comparing them between BH and free-breathing (FB) conditions during a pTx calibration scan in healthy volunteers at 7T.

## Materials and Methods

### Subjects

From August 2015 to October 2015, nine healthy volunteers were examined. All volunteers [seven men and two women; age, 29–50 years (mean age, 36.4 years)] were confirmed to have no neurological signs and symptoms. All study protocols were approved by the local ethics committee and informed consent was obtained from all volunteers before this study.

### MRI

A 7T MRI scanner (Discovery MR950; GE Healthcare, Waukesha, WI, USA) was used with a two-channel quadrature head coil (Nova Medical, Wilmington, MA, USA). The RF transmission system had two individual RF channels (i.e. Ch0 and Ch1) in which the amplitudes, phases, and shape of RF were independently controlled. The safety of pTx scanning (i.e., RF shimming and RF design) were assured that all scans were only single slice and performed under one-tenth of the specific absorption rate (SAR) limit in the first-level controlled operation mode to ensure a sufficient safety margin.

### Calibration scans for pTx

The calibration scan for pTx was performed using a GRE sequence with a repetition time (TR) of 33 ms, echo time (TE) of 8.3 and 9.2 ms, flip angle (FA) of 20 degrees, matrix size of 32 × 32, and section thickness of 5 mm. An off-resonance pulse was applied immediately following excitation in order to obtain magnitude and phase images, and B1+ maps of each RF channel were created from two phase images based on the Bloch-Siegert method.^11^ The duration of the off-resonance pulse was 4.0 ms and the frequency shift was ±4 kHz relative to water. B0 maps were also obtained with phase images of two different TEs (8.3 and 9.2 ms) in the same scan.^16^ The total scan time of this calibration scan was 13 s.

These calibration scans were conducted under FB and BH conditions to evaluate the effect of breathing. BH was performed in the expiratory phase under respiratory monitoring using abdominal bandage.

### Evaluation of image signal intensities

After the pTx calibration, a single section scan of GRE image (2D-gradient-recalled-acquisition in steady state) was performed under FB, with a 24-cm field of view, section thickness of 5 mm, number of excitation (NEX) of 1, repetition time (TR) of 300 ms, echo time (TE) of 10 ms, FA of 10 degrees, and matrix size of 256 × 128. The acquisition time was 42 s, and the section location was set at the level of the basal ganglia.

We used two different modes of pTx for these GRE scans; RF shimming and RF design. In this study, “spoke method” was used for RF design, with B0 and B1+ maps included in the calculation.^6,7^ The number of spokes was fixed to three. As one spoke has been reported to be equivalent to RF shimming,^17,18^ we used one-spoke as RF shimming, with only B1+ map included in the calculation.

In addition to these two pTx scans, a qTx scan was also added for comparison, in which the same amplitude and orthogonally different phases between the two RF channels were used. Thus, six types of scans; session A; qTx, RF shimming FB, RF shimming BH, session B; qTx, RF design FB, and RF design BH were performed in one session. This session (A and B) was repeated respectively five times (five sessions) to examine the reproducibility.

### Region-of-interest (ROI) measurements and statistical comparisons

**Fig 1.**
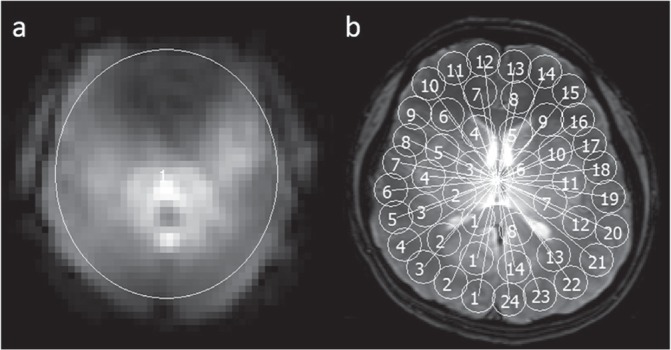
Examples of region-of-interest (ROI) analysis. For the evaluation of B0 and B1+ maps, a single ROI is placed to cover the whole brain (**a**). In contrast, ROIs with a diameter of 9.4 mm (i.e., 10 pixels) are automatically placed using in-house software for the evaluation of gradient echo images (**b**).

The ROI measurements were performed on B0 and B1+ maps of each channel, and the GRE image of pTx was obtained using in-house software [perfusion mismatch analyzer (PMA), which is available at the Acute Stroke Imaging Standardization Group—ASIST-Japan website at http://asist.umin.jp]. A single oval ROI was manually placed on the magnitude image of calibration scans covering the whole brain (Fig. 1a), and average B0 and B1+ values in the ROI were measured. The ROIs of GRE images were automatically placed concentrically in the brain with a diameter of 9.4 mm (i.e., 10 pixels) and the total numbers of ROIs in each subject were 47–50 (mean, 49.2) (Fig. 1b).

The reproducibility of B0 and B1+ maps was evaluated by determining the inter-session standard deviation (SD), which reflected variations in ROI values among five sessions in each volunteer. The average SD values of all volunteers were compared between FB and BH with the Wilcoxon’s sum rank test.

The reproducibility of GRE images was also evaluated by determining inter-session coefficients of variation (CV), in which the CV was calculated by determining the SD/average over five sessions in each location in each volunteer. The average CVs of all locations of all volunteers were compared among FB, BH, and qTx, with Dwass-Steel-Critchlow-Fligner procedure.

Finally, the signal inhomogeneity in each GRE image was evaluated by in-plane CV measurements. The average and SD values of all ROIs in one image were measured, and CV was calculated for each GRE image. Then, average CVs of all the sessions in all the volunteers were also compared among FB, BH, and qTx with the Dwass-Steel-Critchlow-Fligner procedure.

All statistical tests were conducted with JMP 12 software (SAS Institute Inc., Cary, NC, USA). The statistical significance was set at a *P* value of less than 0.05.

## Results

**Fig 2.**
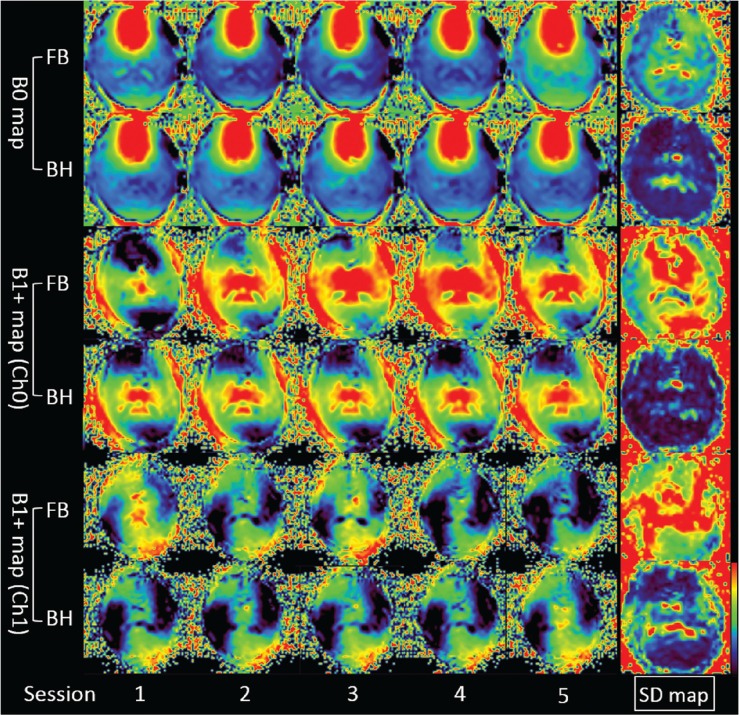
B0, B1+, and SD maps of one volunteer. Five sessions of B0 and B1+ maps are shown for FB and BH, and the SD maps calculated from those five maps are also shown. The window level and width of these images are the same in B0, B1+, and each SD maps, respectively. Note that B1+ maps are created for each of two channels. B0 maps vary across five sessions in FB (top row), while those are constant in BH (second row). B1+ maps of each channel also show variation across five sessions in FB (third and fifth row), and those maps are constant in BH (fourth and sixth row). As a result, values of SD maps are higher in FB than in BH, both for B0 and B1+ maps, indicating that reproducibility is better in BH than in FB. FB, free-breathing; BH, breath-holding; SD, standard deviation.

Figure 2 shows typical examples of B0 and B1+ maps for five sessions, and their SD maps. The B0 maps in FB showed a large frequency shift at the frontal region of the brain, probably due to the air in the frontal sinus. This frequency shift was consistent in all scans for both FB and BH; however, the frequency shift in other parts of the brain varied among the five sessions in FB, whereas the variations among five sessions were smaller in BH. These differences in frequency variations between FB and BH were obvious in SD maps, which showed variations among sessions. In addition to the B0 map, the B1+ maps of each channel (i.e., Ch0 and Ch1) in FB also showed inter-session variations. These variations were lower in BH, and the values in the SD maps were lower in BH than in FB.

**Fig 3.**
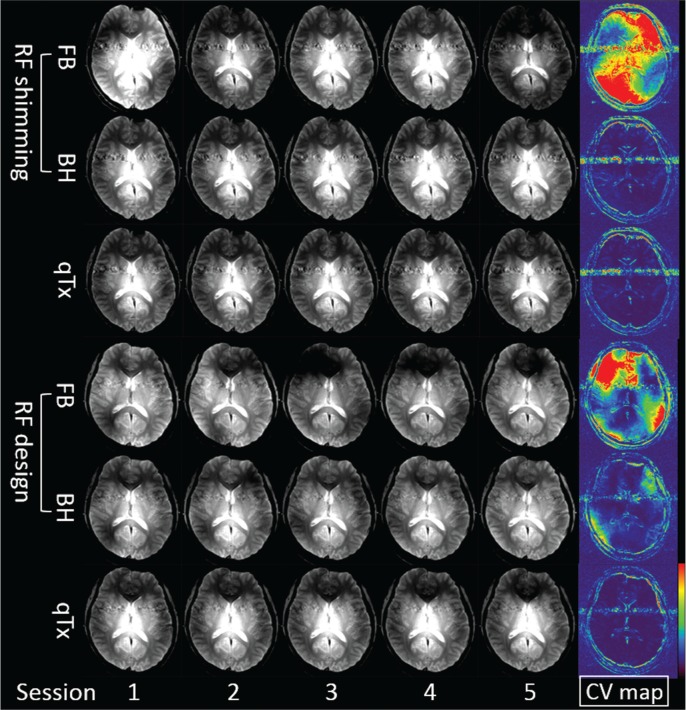
GRE images of one volunteer. Five sessions of GRE images in the same volunteer as in Fig. 2 are shown for FB and BH of pTx (RF shimming and RF design), as well as qTx. The window level and width of these images are the same in GRE images and CV maps, respectively. CV maps calculated from those five images are also shown. GRE images show variations across five sessions with FB (top and fourth row), while they are constant for BH (second and fifth row), both for RF shimming and RF design. Images are also constant in qTx (third and sixth row). As a result, CV maps have higher values in FB compared to BH and qTx, indicating a better reproducibility in BH than in FB. Note that the high-intensity area in the center of brain in qTx is better in RF design. BH, breath-holding; CV, coefficient of variation; FB, free-breathing; GRE, gradient echo; pTx, parallel transmission; qTx, quadrature transmission. RF, radiofrequency.

Figure 3 shows examples of GRE images for five sessions in the same volunteer, and the corresponding CV maps. The GRE images of the qTx showed high-intensity signals in the center of the brain, whereas low-intensity signals were noted in the peripheral area, especially in the frontal area. The qTx images were similar in all five sessions, and the values in the CV maps were homogeneously low, indicating that qTx had good reproducibility. With RF shimming, the high signals in the center of brain were still obvious in both FB and BH. In addition, there were significant changes in the distribution of signal inhomogeneities through five sessions in FB, and the CV map showed high values at the roughly two diagonal regions. In contrast, however, those fluctuations among sessions were considerably better in BH, and CV maps were similar to qTx. With the RF design, high signals in the center of brain were not so obvious compared to qTx. However, there were significant fluctuations among sessions in FB, and severely low signals were noted in a number of sessions. The CV map in FB showed irregular-shaped high values at random areas. In contrast, the fluctuations among sessions became smaller in BH, and the CV map showed low values in most areas of the brain.

**Fig 4.**
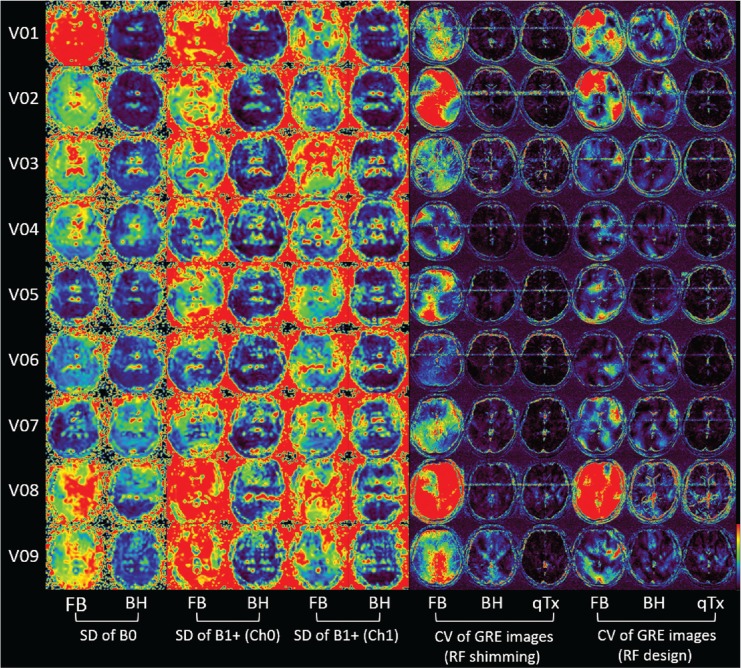
SD maps of B0 and B1+ maps, and CV maps of GRE images of all volunteers. The values of the SD maps of B0 and B1+ maps of each channel are higher in FB than BH in all the volunteers. The window level and width of these images are the same in SD maps of B0 and B1+, and CV of GRE images, respectively. The CV maps of GRE images also showed higher values in FB than BH, both for RF shimming and RF design. The values in CV maps are also small in qTx. BH, breath-holding; CV, coefficient of variation; FB, free-breathing; GRE, gradient echo; RF, radiofrequency; SD, standard deviation.

The SD maps for B0 and B1 maps and the CV maps for GRE images in all the volunteers are summarized in Fig. 4. In all the volunteers, the values in these SD and CV maps were smaller in BH than in FB.

### ROI analysis

**Fig 5.**
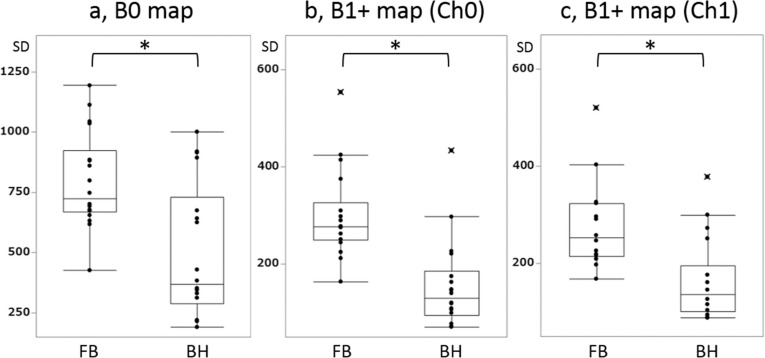
Inter-session standard deviation (SD) of B0 and B1+ maps. The inter-session SD of B0 maps is significantly lower in BH than FB (**a**, *P* < 0.01). For each channel of B1+ maps, SDs are also significantly lower in BH than FB (**b** and **c**, *P* < 0.01 for both). ^*^P < 0.01; BH, breath-holding; FB, free-breathing.

In the B0 map, inter-session SDs of BH (mean, 499.5; range, 191.1–1000.4) were significantly smaller than those of FB (mean, 794.7; range, 426.4–1192.5) (*P* < 0.05, Fig. 5A). In the B1+ maps, the CVs of BH (Ch0: mean, 155.7, range, 70.9–433.3; Ch1: mean, 163.0, range, 88.3–377.9) were also significantly smaller than those of FB (Ch0: mean, 301.5, range, 163.9–553.3; Ch1: mean, 272.8, range, 168.4–553.3,) (*P* < 0.01 for both, Fig. 5B), suggesting that the reproducibility of the B0 and B1+ maps was improved by BH.

**Fig 6.**
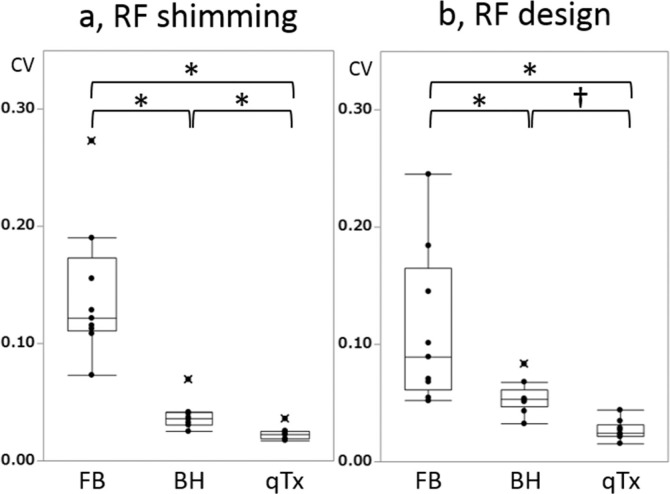
Inter-session coefficients of variance (CV) of GRE images. In RF shimming, the inter-session CV is significantly lower in BH than FB (**a**, *P* < 0.01). The CV is also significantly lower in BH than FB in RF design (**b**, *P* < 0.05). Note that CVs of qTx are smallest and significantly different to FB and BH, both for RF shimming and RF design. ^*^P < 0.01, ^†^*P* < 0.05; BH, breath-holding; FB, free-breathing; GRE, gradient echo; RF, radiofrequency; qTx, quadrature transmission; RF, radiofrequency.

For the GRE images, the inter-session CVs of RF shimming were significantly larger in FB (mean, 0.142; range, 0.073–0.273) than in qTx (mean, 0.023; range, 0.017–0.036) (*P* < 0.01, Fig. 6A). When BH was applied, CV became significantly lower (mean, 0.038; range, 0.025–0.069) than FB (*P* < 0.01); however, the CV of BH was slightly but significantly larger than that of qTx (*P* < 0.01). In RF design, the inter-session CV was also significantly larger in FB (mean, 0.112; range, 0.052–0.245) than qTx (mean, 0.027; range, 0.016–0.044) (*P* < 0.01, Fig. 6B). CV also became significantly smaller in BH (mean, 0.054; range, 0.032–0.083); however, it was still significantly different from qTx (*P* < 0.05).

**Fig 7.**
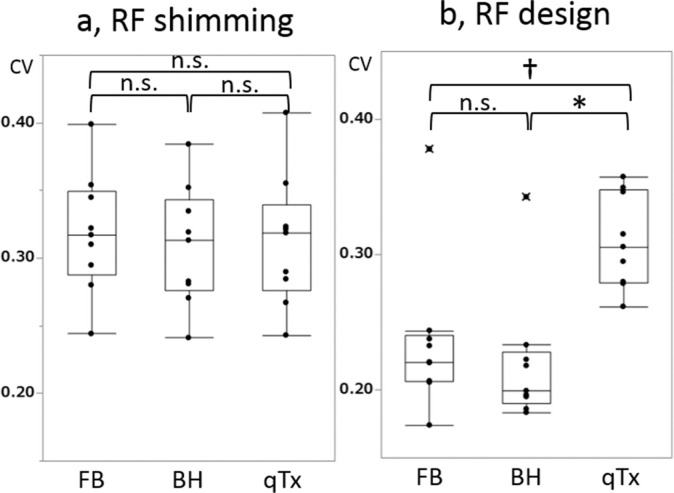
In-plane coefficients of variance (CV) of GRE images. In RF shimming, the in-plane CVs are not significantly different among FB, BH, and qTx (**a**). In contrast, in-plane CVs of FB and BH are significantly smaller than in qTx (**b**, *P* < 0.01 and *P* < 0.05, respectively). The CVs are not significantly different between FB and BH. ^*^P < 0.01, ^†^*P* < 0.05; BH, breath-holding; FB, free-breathing; GRE, gradient echo; qTx, quadrature transmission; RF, radiofrequency.

In RF shimming, in-plane CVs for both FB and BH (mean, 0.318, range, 0.244–0.399; and mean, 0.309, range, 0.241–0.382, respectively) were not significantly different with qTx (mean, 0.312; range, 0.243–0.407) (qTx vs. FB; *P* = 0.983, and qTx vs. BH; *P* = 0.962), suggesting that signal inhomogeneity was not improved by RF shimming (Fig. 7A). In contrast, the in-plane CVs of the RF design were significantly lower in FB (mean, 0.235; range, 0.174–0.378) and BH (mean, 0.220; range, 0.183–0.342) than in the qTx (mean, 0.310; ranged, 0.262–0.357) (qTx vs. FB; *P* < 0.05, qTx vs. BH; *P* < 0.01), and there was no significant difference between FB and BH (*P* = 0.485) (Fig. 7B).

## Discussion

In this study, we found that reproducibility in B0 and B1+ mapping using the Bloch-Siegert method at 7T was improved by BH, suggesting that these mappings were sensitive to respiratory motion. In addition, the clinical images of the GRE sequence also showed respiratory fluctuations when pTx of RF shimming or RF design was used, and these fluctuations were improved by BH during the calibration scans for B0 and B1+ mapping. Although the reproducibility of both RF shimming and RF design were slightly worse than in qTx, we found that RF design had better signal homogeneity than qTx. Therefore, in our study, RF design with BH during calibration scans was the best combination to achieve homogeneous and reproducible images with pTx in 7T MRI.

Inhomogeneity in the transmit RF field was not a serious problem before the advent of UHF techniques such as 3T or 7T MRI, because of the smaller resonant frequency in the low field strength. However, in UHF techniques, the larger resonant frequency and the shorter RF pulse cause a strong B1+ inhomogeneity;^1,3,4^ therefore, methods for improving the B1+ inhomogeneity are needed. The postprocessing method for correcting the intensity inhomogeneity at 7T is useful and suitable for practical use in part.^19^ However, there are some problems related to the image quality in cases with a strong local signal loss and huge abnormal-intensity intracranial lesions, thereby emphasizing the signal inhomogeneity and lowering the CNR.^20^ To improve the intensity homogeneity or B1+ homogeneity, previous studies that used 7T demonstrated the development of pTx such as the optimization of the number of coil elements, geometric factors, SENSE factors, RF shimming method, and special image sequences.^18,21–26^ Most of these studies have used a human head model phantom or simulation of a digital human head model, and few volunteers have been used.

The B0 fluctuations caused by respiration have been actively reported in the field of functional MRI. It has been reported that the phase shifts occurred between inspiration and expiration due to the motion of the chest and local oxygen concentration changes, and this effect became larger in the UHF, especially at 7T.^12–15^ Our result showing improvement of B0 fluctuations by BH was consistent with those of fMRI studies. In addition, we also found that the same phenomenon was seen in B1+ mapping with the Bloch-Siegert method, in which two phase images were used with two off-resonance pulses. We assume that respiratory fluctuations of B0 also affect the relative frequency shift induced by those off-resonance pulses, and that might cause respiratory fluctuations on B1+ maps. The B1+ mapping with the Bloch-Siegert method was used commonly because it has been reported to be accurate, efficient, and largely insensitive to T_1_ and T_2_, and it produces high angle-to-noise ratio maps.^11^ The robustness of the Bloch-Siegert method may be based on the fact that the phases were not largely changed by respiration at values below 3T. However, in the UHF, especially at 7T, the phase variation might be large enough to influence the B1+ mapping. To our knowledge, no study has been conducted to study this phenomenon and the reproducibility of the pTx scan. As the B0 and B1+ map is used for the calculation of optimal amplitude and phase in RF shimming, and RF design, the improvement of reproducibility in B0 and B1+ mapping by BH was important for the subsequent image acquisition with pTx. In fact, our results also showed that the variations in GRE images among sessions were improved by BH during the calibration scans of B0 and B1+ mapping, suggesting that reproducibility in B0 and B1+ mapping is important for the reproducibility of GRE images.

Our study demonstrated that RF shimming could not improve the in-plane signal inhomogeneity compared to qTx, while some studies^7,23,25^ demonstrated better signal homogeneity using multi-channel pTx (e.g., eight channels and over) with RF shimming. We assume that the number of pTx channels (two channels in this study) was too small to achieve better signal homogeneity. However, significantly better intensity homogeneity was achieved in RF design compared to qTx in our system. While the RF shimming could only modulate the RF amplitude and phases, RF design has combined modulation of the amplitude, phase, and waveform of RF pulse. Therefore, we assume that RF design successfully improves intensity homogeneity even with a two-channel head coil.

There are several limitations in our study. First, BH was applied only during the calibration scans, not during the GRE scans. As all the GRE images were acquired with FB, the respiratory B0 fluctuations could arise during the scan, which was different in the calibration scan. As the duration of GRE sequence we used was 43 s, we could not apply BH in GRE imaging. The breathing during GRE scans could cause B0 fluctuations and it may be a reason that the signal reproducibility in the RF design was slightly worse than that in the RF shimming, in which a B0 map was not required for the calculation of RF. Respiratory triggering might be effective to further improve the reproducibility in RF design. Second, the rhythm and depth of respiration, and the type of respiration (e.g., abdominal or chest) was variable among volunteers. These variations in respiration may affect the degree of respiratory fluctuations. Third, the B0 mapping was conducted by two phase images scanned separately with different TEs in this study, and these two phase images may be influenced by the different respiratory status. This might be a cause of inaccuracy in B0 mapping. Fourth, only GRE sequences were tested because of the system limitations of our 7T system. For clinical purposes, spin echo (SE) or fast spin echo (FSE) sequences are frequently used in addition to the GRE sequences. The SE sequence consists of two different FAs of RF pulse. A higher FA may lead to a stronger signal inhomogeneity, compared to the lower FA in the GRE sequence. Further studies using SE and FSE sequences are needed to compare pTx and qTx. Fifth, we performed our evaluations using only single-section sequences because of the limitation of our 7T system. For clinical use, it may be necessary to perform evaluations using multisection sequences.

## Conclusion

In conclusion, BH during calibration scans could improve the reproducibility of B0 and B1+ mapping in comparison with FH, and could improve the signal reproducibility of GRE images in both RF shimming and RF design in the human brain. Additionally, the RF design of pTx was better than RF shimming with regard to improvement of signal homogeneity, in comparison with qTx. These results might facilitate the development of pTx in 7T MRI for humans.
